# A novel method for screening malignant hematological diseases by constructing an optimal machine learning model based on blood cell parameters

**DOI:** 10.1186/s12911-025-02892-1

**Published:** 2025-02-11

**Authors:** Dehua Sun, Wei Chen, Jun He, Yongjian He, Haoqin Jiang, Hong Jiang, Dandan Liu, Lu Li, Min Liu, Zhigang Mao, Chenxue Qu, Linlin Qu, Ziyong Sun, Jianbiao Wang, Wenjing Wu, Xuefeng Wang, Wei Xu, Ying Xing, Chi Zhang, Jingxian Zhang, Lei Zheng, Shihong Zhang, Bo Ye, Ming Guan

**Affiliations:** 1 Department of Clinical Laboratory, Nanfang Hospital, Guangzhou, 516006 China; 2 Department of Clinical Laboratory, The First Affiliated Hospital of Xi’an Jiaotong University, Xi’an, 710061, China; 3 Department of Clinical Laboratory, The First Affiliated Hospital of Soochow University, Suzhou, 215006 China; 4 Department of Clinical Laboratory, Huashan Hospital Fudan University, Shanghai, 200040 China; 5 Department of Clinical Laboratory, West China Hospital of Sichuan University, Chengdu, 610044 China; 6 Clinical Department (IVD), Shenzhen Mindray Bio-Medical Electronics Co, Ltd, Shenzhen, 518057 China; 7 Department of Clinical Laboratory, The First Affiliated Hospital, Sun Yat-Sen University, Guangzhou, 510062 China; 8 Department of Clinical Laboratory, Peking University First Hospital, Beijing, 100034 China; 9 Department of Clinical Laboratory, The First Bethune Hospital of Jilin University, Jilin, 130061 China; 10 Department of Clinical Laboratory, Tongji Hospital, Tongji Medical College of Hust, Wuhan, 430030 China; 11 Department of Clinical Laboratory, Ruijin Hospital, Shanghai Jiaotong University School of Medicine, Shanghai, 200025 China

**Keywords:** Artificial intelligence, Blood cell parameters, Malignant hematological diseases, Machine learning

## Abstract

**Background:**

Screening of malignant hematological diseases is of great importance for their diagnosis and subsequent treatment. This study constructed an optimal screening model for malignant hematological diseases based on routine blood cell parameters.

**Methods:**

The venous blood samples of 1751 patients collected from 10 tertiary hospitals in China were divided into a training set (1223 cases) and a validation set (528 cases). In addition to the clinical diagnostic information of the samples in the training set, 26 blood cell parameters including morphological parameters were selected using manual screening and filtering to construct eight machine learning models. These models were used to identify hematological malignancies among the validation set.

**Results:**

Comparison of the discrimination, calibration and clinical detection performance of the eight machine learning models revealed that the artificial neural network (ANN) model performed the optimal in identifying malignant haematological diseases in the validation set (528 cases), with an area under the receiver operating characteristic curve (AUC), accuracy, sensitivity and specificity of 0.906, 0.857, 0.832 and 0.884, respectively.

**Conclusion:**

The ANN model constructed can be used for screening of malignant hematological diseases, especially in primary hospitals that lack comprehensive diagnosis, and this ANN model will help patients to get diagnosis and treatment of malignant hematological diseases as early as possible.

**Supplementary Information:**

The online version contains supplementary material available at 10.1186/s12911-025-02892-1.

## Highlights

• This study is the first to construct eight machine learning models for screening malignant hematological diseases using blood cell parameters.

• Comparison of the discrimination, calibration and clinical detection performance of the eight machine learning models constructed in this study revealed that the artificial neural network (ANN) model had the best performance.

• This is a multicentre study involving up to 10 tertiary hospitals, which avoids data bias and provides convincing data from a diverse population.

**Supplementary Information:**

The online version contains supplementary material available at 10.1186/s12911-025-02892-1.

## Introduction

According to the International Agency for Research on Cancer's 2020 survey on the incidence of 36 types of cancers in 185 countries throughout the world, hematological malignancies (including non-Hodgkin lymphoma, leukemia, multiple myeloma and Hodgkin lymphoma) were experienced by more than 1 million new patients in 2020 and resulted in more than 600,000 new deaths [1]. This group also found that due to the growth and aging of the population, the incidence of cancer is expected to increase by 47% in 2040, potentially imposing enormous burdens on society and the economy and highlighting the need for the early prevention, diagnosis and treatment of these cancers [1, 2]. Due to the diverse and insidious clinical manifestations of the abovementioned malignant hematological diseases, whose clinical diagnoses are particularly challenging, the World Health Organization (WHO) and the National Comprehensive Cancer Network have developed a series of identifying and classification tests, including peripheral blood count and morphological confirmation, bone marrow aspiration and biopsy, imaging studies, immunophenotyping, cytogenetic testing, lymph node biopsy, and serum protein testing [3–5].

Among the above proposed examinations, peripheral blood count analysis and morphology are the easiest to perform and the least expensive. In addition, due to improvements in automated blood cell analyzer technology, the Mindray BC-7500 CRP not only provides the parameters on the report card, but also uses laser flow cytometry in conjunction with scatter fluorescence cube (SF cube) technology to collect a large amount of cellular information, such as volume or intracellular complexity, which can be further converted into study parameters (e.g. total nucleated cell—WNB (TNC- N), neutrophil-to-lymphocyte ratio (NLR)). [6]. The automatic cell morphology analyzer MC-80 can detect the morphology of peripheral blood. It can take high-definition images of up to 20 slices at a time with a 1000 × lens through depth-of-field fusion. The images can be preclassified based on artificial intelligence (AI) [7]. The MC-80 and BC-7500 CRP can comprehensively analyze the peripheral blood cell characteristics of samples. The large amount of blood cell parameter data makes it difficult for professional technicians to distinguish the subtle differences between different diseases. With the increasing use of AI in the medical field, existing research has established a machine learning (ML) model to identify relevant blood cell parameters in sepsis and blood system-related diseases, thereby allowing initial disease screening [8–11]. Based on the above, this study will combine all the parameters output by a BC-7500 CRP auto hematology analyzer and an MC-80 automated morphology analyzer to comprehensively present the whole picture of blood cells in patients with malignant hematological diseases (lymphoma, leukemia, multiple myeloma and MDS).

In recent years, ML and AI have been increasingly used in various branches of medicine [12]. For example, Logistic regression (LR), Naïve Bayes (NB), K-nearest neighbor (KNN), Support vector machines (SVM), Random Forest (RF), Multi-layer perceptron (MLP), Gradient Boosting Decision Tree (GBDT) and Artificial Neural Networks (ANN) models have been applied in auxiliary disease diagnosis and prognosis prediction [8, 13, 14]. In 2020, Shabbir used cell population data (CPD) to establish an ANN model for preliminary screening malignant hematological diseases, achieving an accuracy of up to 0.828 [13]. However, according to the Transparent Reporting of a Multivariable Prediction Model for Individual Prognosis Or Diagnosis (TRIPOD) statement and recent studies, in addition to demonstrating its discriminability (with metrics including accuracy, receiver operating characteristic curve (ROC) and precision-recall curve), basic information about ML models should also be presented in terms of calibration and clinical screening efficacy [15, 16]. This study comprehensively evaluated the performance of eight ML models in terms of discrimination, calibration, and clinical detection efficacy according to the TRIPOD guidelines and selected the best predictive model to further analyze its predictive black-box efficacy.

## Methods

### Study design

**Fig. 1:**
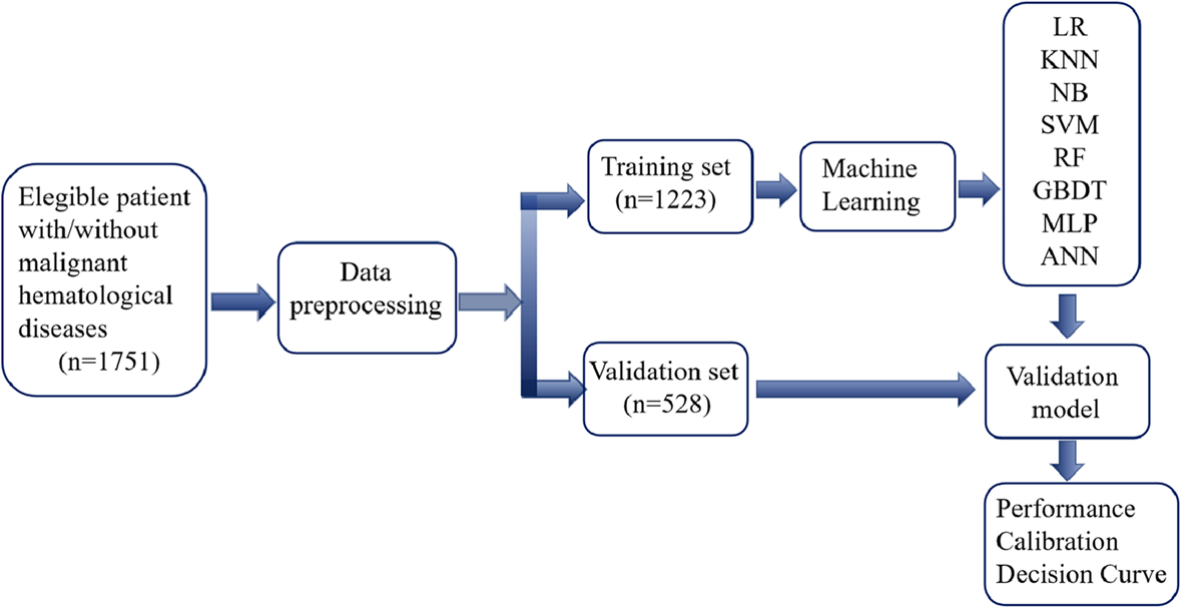
Flowchart of model building. LR: Logistic regression; KNN: K-nearest neighbour; NB: Naïve Bayes; SVM: Support vector regression; RF: Random Forest; MLP: Multiple layer perceptron; GBDT: Extreme Gradient Boosting; ANN: Artificial Neural Network

In this study, the venous blood samples and case information of 1751 patients who triggered routine blood re-examination rules at Nanfang Hospital, The First Affiliated Hospital of Xi’an Jiaotong University, Huashan Hospital Fudan University, The First Affiliated Hospital of Soochow University, West China Hospital of Sichuan University, The First Affiliated Hospital of Sun Yat-sen University, Peking University First Hospital, The First Bethune Hospital of Jilin University, Tongji Hospital and Ruijin Hospital were collected from March to May 2022 [17]. The venous blood samples were subjected to blood cell analysis on a Mindray BC-7500 CRP (Mindray, Shenzhen, China) and blood cell morphology analysis on an MC-80 (Mindray, Shenzhen, China). Then, the analysis results were recorded, and the patient's disease information was organized according to the International Classification of Diseases (ICD-10). The entire process of the study is shown in Fig. 1. All patients in this study underwent a complete diagnostic examination, in which the diagnosis of leukemia and related hematological diseases was identified, confirmed, and classified by a series of tests established by the WHO [3]. This study was approved by the Ethics Committee of Nanfang Hospital [NFCC-2022–352].

### Data preprocessing

Among the 1751 patients enrolled in this retrospective study, the percentage of male and female were 53.4% and 46.6%, respectively. The median age of the male patients was 48 years, and the median age of the female patients was 50 years. The blood cell analysis results of the collected 1751 samples were preprocessed, mainly via data cleaning and normalization. Data cleaning was performed mainly to check for outliers, duplicate values and incorrect inputs in the data and address with them accordingly to ensure that the data quality met the modeling requirements. Normalization was performed because in the original data collected in this study, differences in the feature sources and measurement units could result in a wide distribution of values. When calculating the Euclidean distances between different samples, features with a large value range would bias the performance of the model. Therefore, to normalize our dataset, we used min–max scalar as the scaling function and normalized each dimension feature to the same interval (0,1). Finally, the preprocessed patient information was divided into a training set and a validation set at a ratio of 7:3.

### Feature selection

A total of 114 parameters were obtained for each sample analyzed on the BC-7500 CRP and MC-80 (93 and 21 parameters, respectively). First, we removed the less commonly used channels and 59 parameters with linear relationships through manual screening, such as the RET channel-related parameters RBC-O, RET, and PLT-O, and then we used Filter to calculate the relationship between the remaining 55 parameters and malignant hematological diseases. Twenty-six parameters with strong correlations with malignant hematological diseases were selected to build the ML models, among which red blood cell volume distribution width-standard deviation (RDW-SD), red blood cell distribution width-coefficient of variation (RDW-CV), white blood cell count (WBC), neutrophil percentage (Neu%), immature granulocyte percentage (IMG%), basophil percentage (Baso%), eosinophil percentage (Eos%), monocyte percentage (Mon%), lymphocyte percentage (Lym%), total nucleated cell count-WNB (TNC-N), red blood cell count (RBC), impedance channel platelet count (PLT-I), plateletcrit (PCT), hemoglobin concentration (HGB), mean corpuscular hemoglobin content (MCHC), mean corpuscular volume (MCV), hematocrit (HCT) and mean platelet volume (MPV) were obtained from the BC-7500 CRP. Monocytes, blasts, segmented neutrophils, neutrophils, basophils, abnormal lymphocytes, immature granulocytes, and primitive cells were obtained from the MC-80.

### Construction of eight ML models

The 26 parameters screened above in this study were used to construct ML models, based on the LR, NB, KNN, SVM, RF, MLP, GBDT and ANN algorithms. The first 7 models were obtained from the Scikit-Learn library with default parameter values, while the ANN was obtained with the Keras model library.

The ANN consists of a 2-layer structure, optimized with the grid search method [18], The batch size was 96 after 300 epochs of training. The first hidden layer has 16 nodes with a ReLU activation function, and the second hidden layer has 48 nodes with a ReLU activation function. The output layer uses a single node with a sigmoid activation function. The output layer provides the prediction of the model based on the input values as a continuous variable ranging from 0 (nonmalignant hematological disease) to 1 (definite malignant hematological disease).

### Evaluating the performance of eight ML models

In this study, 528 samples from the validation set were used to evaluate the eight ML models in terms of discriminability, calibration efficiency, and clinical applicability [15, 16]. 1. Discriminability reflects the ability of the ML model to screen malignant hematological diseases from samples that could trigger peripheral blood re-examination. This study assessed the performance of the different ML models when given a default threshold, the thresholds and performance of the different ML models when maximizing the area under the ROC curve (AUC) and the threshold and performance of the different ML models when the precision and recall rate were maximized (i.e., precision-recall curve), to select the ML models that can achieve excellent classification performance from different perspectives. 2. Calibration reflects the consistency between the predicted and actual risk of malignant hematological disease of the different ML models. The differences between the ML models were mainly observed by comparing their calibration curves/Brier scores. ML models with good discriminability were further calibrated with Platt scaling. 3. Clinical applicability for the different ML models is reflected through decision curve analysis (DCA). In addition to the above evaluations, we used confusion matrices to observe sample cases where the predicted results of the ML model did not match the actual results and further analyze the reasons. We also used the Shapley Additive Explanation (SHAP) Python package (version 0.40.0) to address the black-box problem with respect to the predictions of the ML models. The rationale behind SHAP is to provide interpretability and transparency in ML models, while its principle is based on the Shapley value from game theory to quantify the contribution of each feature to the model's prediction. This makes SHAP a powerful tool for understanding and explaining complex models, enhancing trust and reliability in their predictions.

This study used Python 3.7.0, Scikit-learn 0.19.2 and Keras 2.5.0 for statistical analysis. *p* value < 0.05 was considered to indicate statistical significance.

## Results

### Basic patient characteristics

**Fig. 2:**
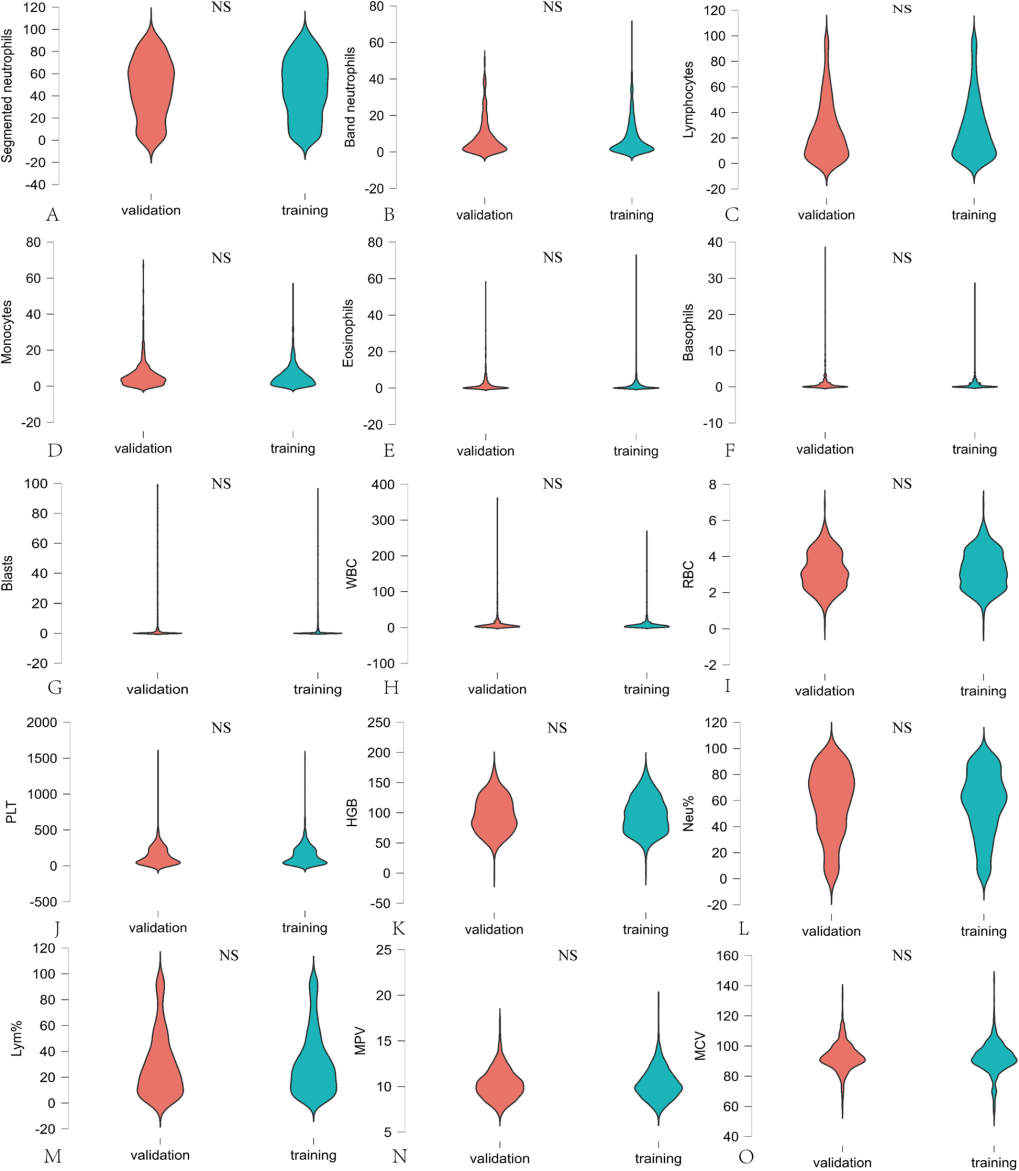
Wilcoxon rank-sum test to analyze parameters of the blood cells in the training and validation sets. **A**, **B**, **C**, **E**, **F**, **G** are all blood cell parameters of MC-80, which are segmented neutrophils, band neutrophils, lymphocytes, monocytes, eosinophils, basophils, blasts. **H**, **I**, **J**, **K**, **L**, **M**, **N**, **O** are all blood cell parameters of BC-7500CRP, which are WBC, RBC, PLT, HGB, Neu%, Lym%, MPV, and MCV

A total of 1751 patients were enrolled in this study; detailed information on their diseases is shown in Table 1. After preprocessing the blood cell analysis results of the 1751 patients, they were divided into a training set and a validation set at a ratio of 7:3. The rank-sum test was used to compare the blood cell parameters between the two datasets, but no significant differences were identified (Fig. 2).

**Table 1:** Case numbers analyzed in the study

ICD-10 Code	Type	Sample size	Label
C81-C96	Malignant neoplasms of lymphoid, haematopoietic and related tissue	910	1
D50-D53	Nutritional anemia	50	0
D55-D59	Haemolytic anemia	38	0
D60-D64	Aplastic and other anemia	33	0
	Other diseases	720	0

Other diseases hematological neoplasms

### Discriminability of the ML Models

**Fig. 3:**
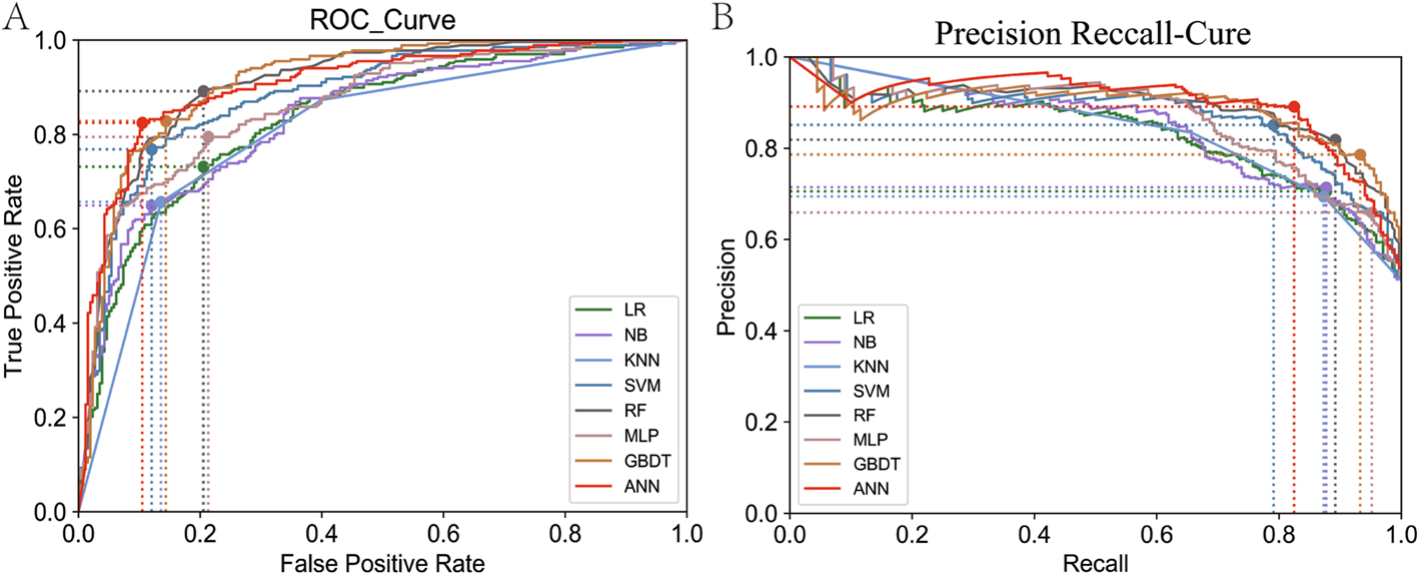
Model performance in the validation set. **A**. Receiver operating for screening malignant haematological diseases patients in the validation set. **B**. Precision-recall curves for screening malignant haematological diseases patients in the validation set. LR: Logistic regression; KNN: K-nearest neighbour; NB: Naïve Bayes; SVM: Support vector regression; RF: Random Forest; MLP: Multiple layer perceptron; GBDT: Extreme Gradient Boosting; ANN: Artificial Neural Network

In this study, 26 blood cell parameters were obtained by feature selection in the construction of the eight ML models, and then the validation set samples were used to verify the ability of each model to distinguish malignant hematological diseases (Supplementary Table 1). Table 2 shows the performance indicators of each model for three different thresholds: a threshold of 0.5, the threshold that maximized the AUC, and the threshold that maximized the F1 score. At each threshold, the AUCs of the eight ML models were all greater than 0.8; specifically, those of ANN, RF and GBDT were all greater than 0.9, and the ANN model had the highest accuracy and F1 score (Fig. 3). Compared with other ML models, the ANN threshold had a smaller threshold range; additionally, its accuracy, precision, sensitivity and specificity were 0.857, 0.881, 0.832 and 0.884, respectively (Table 2).

**Table 2:** The performance measures evaluated for different ML and AI methods for screening malignant haematological diseases use different thresholds found in the validation set

Model	Thresholds	AUC	Accuracy	Precision	Sensitivity	Specificity	F1-score
**The thresholds of 0.5**							
LR	0.5	0.834	0.749	0.722	0.825	0.671	0.671
NB	0.5	0.836	0.709	0.897	0.485	0.942	0.942
KNN	0.5	0.809	0.759	0.834	0.657	0.864	0.864
SVM	0.5	0.881	0.808	0.804	0.825	0.791	0.791
RF	0.5	0.907	0.838	0.830	0.858	0.818	0.818
MLP	0.5	0.864	0.785	0.786	0.795	0.775	0.775
GBDT	0.5	0.913	0.831	0.823	0.851	0.810	0.810
ANN	0.5	0.906	0.857	0.881	0.832	0.884	0.884
**The threshold for the best optimal ROC Youden index**							
LR	0.602	0.834	0.760	0.786	0.728	0.795	0.795
NB	0.000	0.836	0.760	0.848	0.646	0.880	0.880
KNN	1.000	0.809	0.490	/	0.000	1.000	1.000
SVM	0.646	0.881	0.821	0.869	0.765	0.880	0.880
RF	0.440	0.907	0.842	0.818	0.888	0.795	0.795
MLP	0.515	0.864	0.789	0.794	0.791	0.787	0.787
GBDT	0.614	0.913	0.840	0.857	0.825	0.857	0.857
ANN	0.522	0.906	0.857	0.891	0.821	0.895	0.895
**The threshold for the best optimal precision-recall**							
LR	0.446	0.834	0.749	0.705	0.873	0.620	0.620
NB	0.000	0.836	0.757	0.713	0.873	0.636	0.636
KNN	0.500	0.809	0.759	0.834	0.657	0.864	0.864
SVM	0.615	0.881	0.821	0.851	0.787	0.857	0.857
RF	0.440	0.907	0.842	0.818	0.888	0.795	0.795
MLP	0.232	0.864	0.722	0.658	0.948	0.488	0.488
GBDT	0.226	0.913	0.835	0.785	0.929	0.736	0.736
ANN	0.522	0.906	0.857	0.891	0.821	0.895	0.895

#### Calibration efficiency of the ML Models

**Fig. 4:**
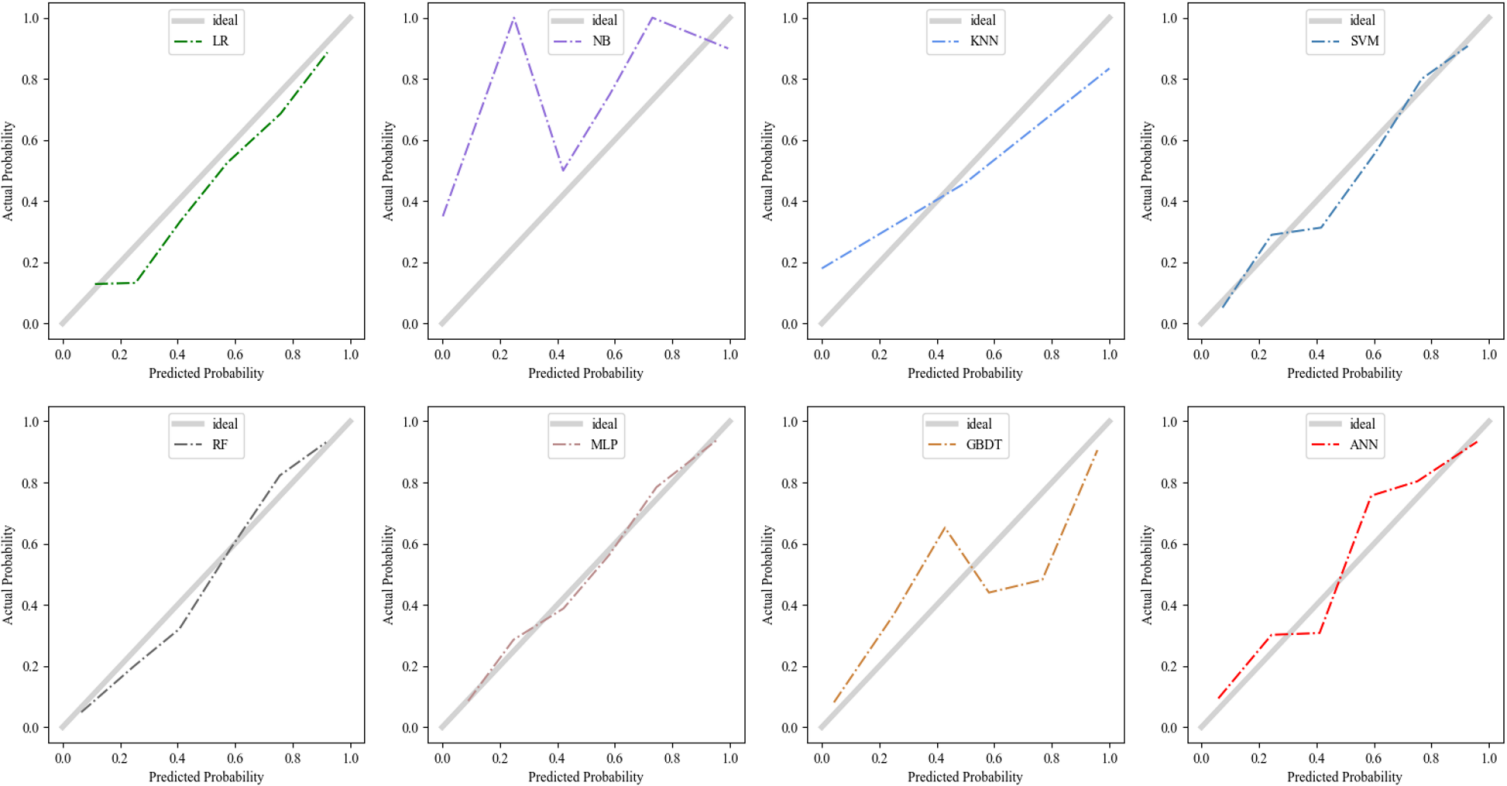
Calibration plots of the ML models for screening malignant haematological diseases patients in the validation set**.** LR, logistic regression; NB,naïve Bayes; KNN, K-nearest neighbour; SVM, support vector regression; RF, Random Forest; MLP, multiple layer perceptron; XGBOOT, extreme gradient boosting

**Fig. 5:**
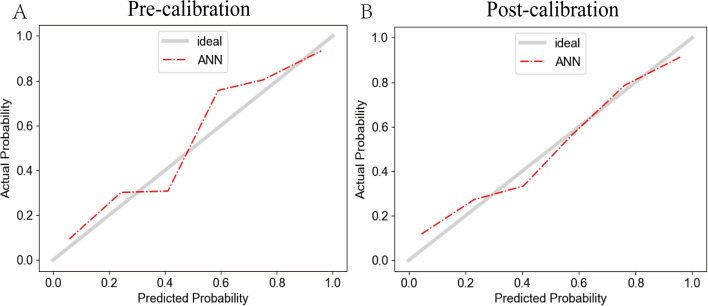
Calibration curves for ANN models. **A**. The ANN model pre-Platt Scalling calibration curves. **B**. The ANN model post-Platt Scalling calibration curves

According to the TRIPOD guidelines [15], calibration is an important part of evaluating ML models. Figure 4 shows the calibration curves for the eight ML models constructed in this study. Note that the calibration curve of the ANN model needs to be further optimized despite its excellent discriminability. Thus, we used Platt scaling to calibrate the ANN model; the results are shown in Fig. 5. There was significant improvement in the agreement between the ANN model prediction of the risk of malignant hematologic disease and the actual proportion of patients with said diseases.

#### Clinical applicability of the ML models

**Fig. 6:**
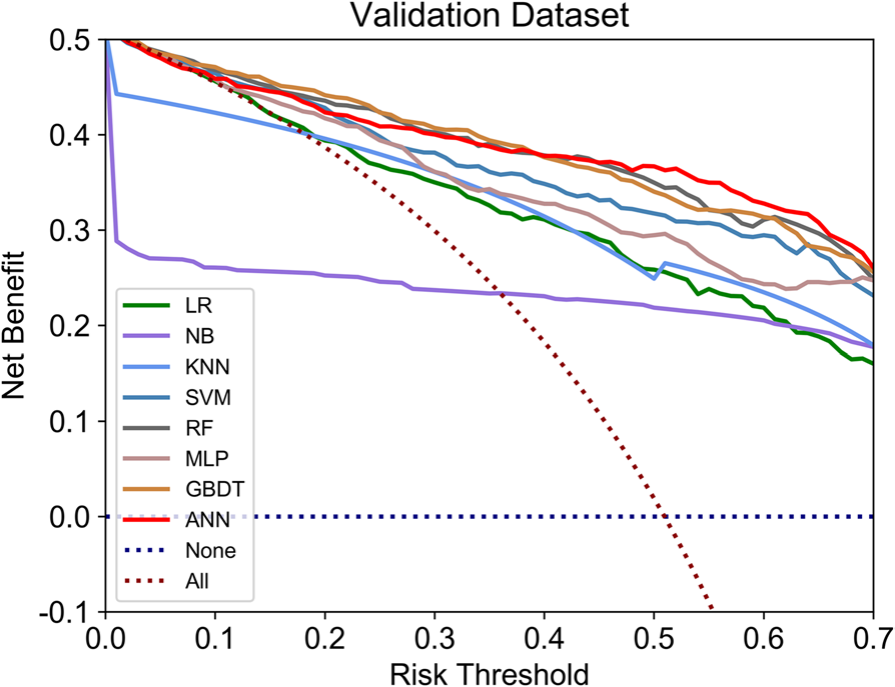
Decision curve analysis. LR, logistic regression; NB, naïve Bayes; KNN, K-nearest neighbour; SVM, support vector regression; RF, Random Forest; MLP, multiple layer perceptron; XGBOOT, extreme gradient boosting

In addition to discrimination and calibration, clinical applicability is another aspect of ML models that needs to be assessed. According to decision curve analysis (Fig. 6), the net clinical benefits of the ANN, RF, and GBDT models were greater than those of other ML models across the range of different thresholds.

#### Analysis of the ANN model

**Fig. 7:**
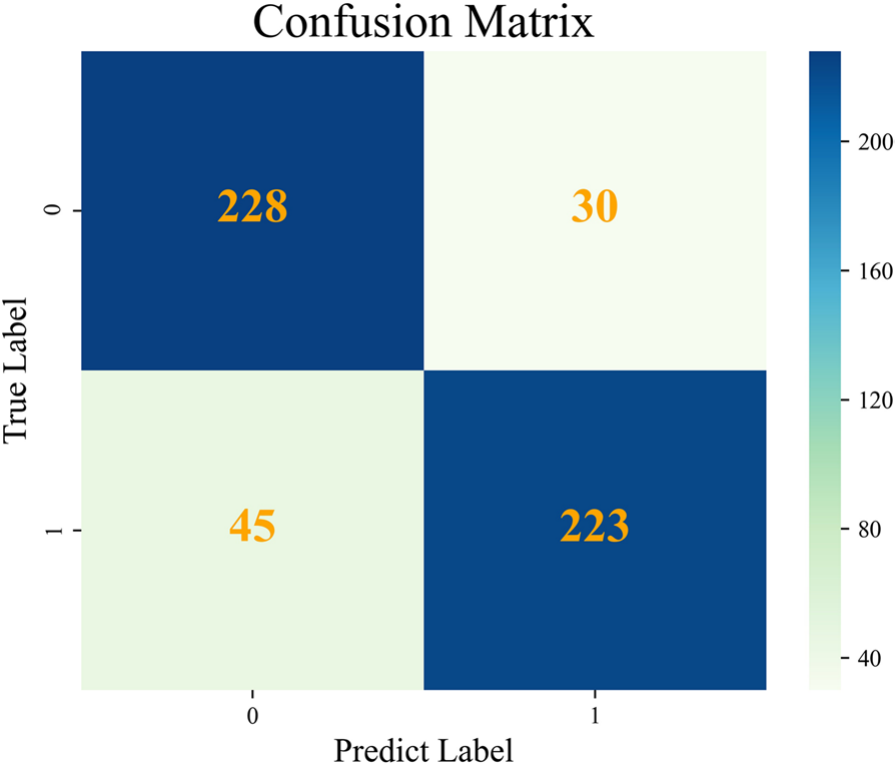
Confusion matrix of the ANN models for screening malignant haematological diseases patients in the validation set

By comparing the performance of the different ML models, we conclude that the ANN model had the best performance in all analyzed aspects. Therefore, we further analyzed the screening efficacy of the ANN model with the 528 samples in the validation set through confusion matrix analysis (Fig. 7). Of the 45 cases classified as false-negative samples, 22 cases were lymphoma and 6 cases were multiple myeloma; the changes in their peripheral blood parameters were not obvious. In addition, 8 patients were being treated for a malignant hematological disease, 7 had acute leukemia, and 2 had MDS. Furthermore, thirty false-positive samples were analyzed, of which 10 were from patients with an infection, 15 were from anemia patients, and 5 were from patients with other diseases.

**Fig. 8:**
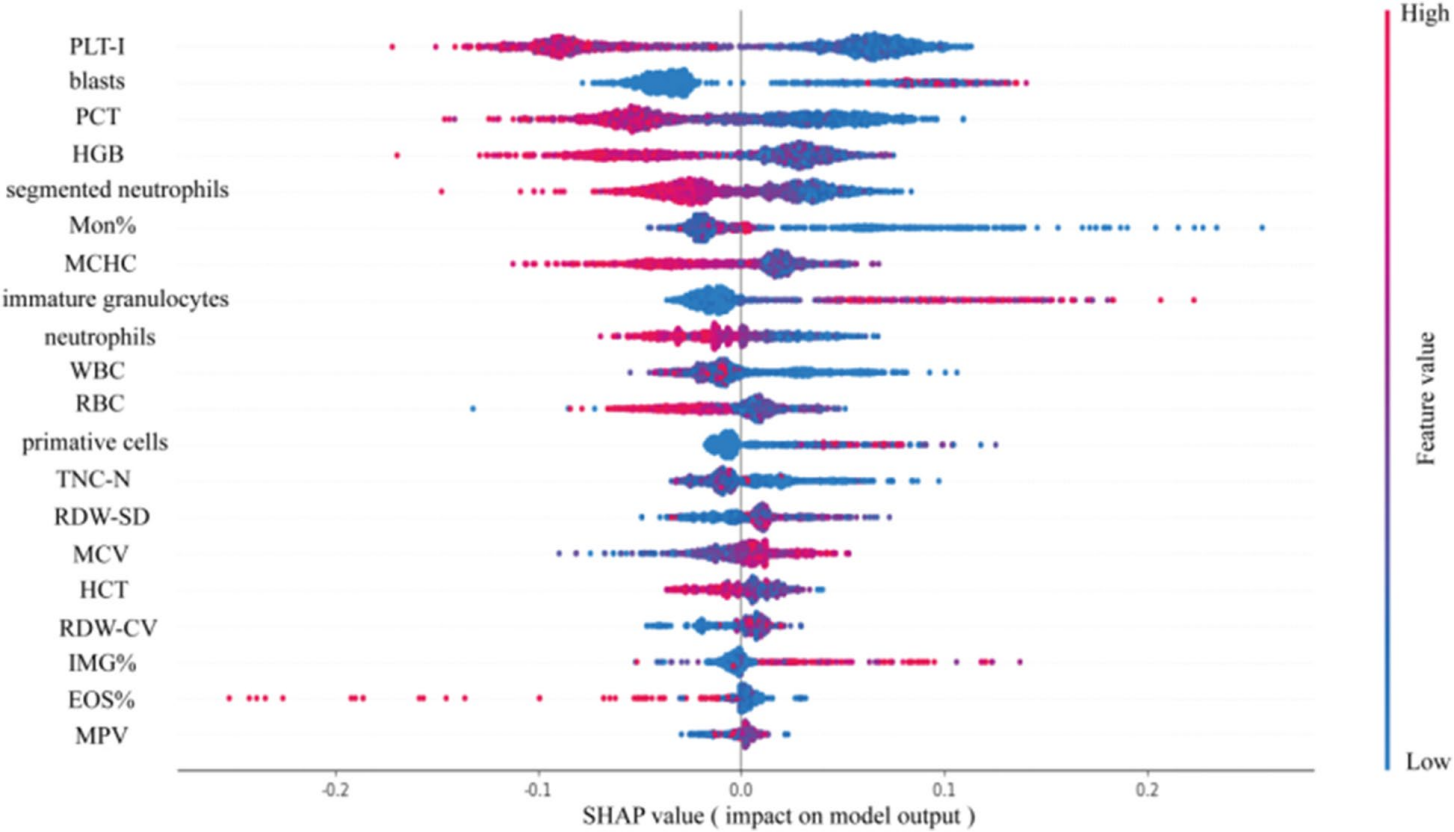
Results of Shapley additive explanation (SHAP) analysis of the ANN model. SHAP summary plot of 20 feature clusters, derived by aggregating related values of a particular feature (e.g., the average, minimum, and maximum). Each dot corresponds to the SHAP value of the feature cluster for the malignant haematological diseases risk score of a given case patient or control subject at a certain point in time. A feature’s SHAP value (x-axis) represents the contribution of the specific feature to the risk score, with positive values indicating a contribution that increases the risk score and negative values indicating a contribution that lowers the score. The location of the dot on the x-axis represents its SHAP value, whereas its color represents the cluster’s value (the actual value of the feature that is represented in the cluster), with red representing higher values (for features measured along a continuum) or affirmative responses (for binary features). The dots are piled up vertically to show their density. The feature clusters are sorted by their mean absolute SHAP values

Next, we sought to address the black-box problem for the ANN model in screening malignant hematological diseases through SHAP analysis, which ranked the features used during model construction according to the feature importance in predicting the status of the samples. Each point represents a sample, with those in red indicating high feature values and those in blue indicating low feature values. Features with positive SHAP values indicated that they are associated with an increased risk of malignant hematological disease (Fig. 8). It can be observed that PLT-I, blasts, and PCT were the most important features in our constructed ANN model.

## Discussion

The clinical manifestations of malignant hematological diseases are mostly increased tumor cells in the blood, bone marrow or lymphoid and other tissues; such diseases include leukemia, lymphoma and multiple myeloma [4]. According to the classification of hematological malignancies formulated by the WHO in 2016, there are more than 60 subtypes of leukemia, myeloma and lymphoma alone. The different manifestations of these numerous subtypes and their long disease courses make both clinical diagnosis and treatment difficult [3, 4]. Peripheral blood may change during different courses of different malignant hematologic diseases [4, 19]. At present, peripheral blood cell analysis not only relies on blood cell analyzers to provide reliable count and classification results but also yields a large number of research parameters. Automatic cell morphology analyzers can also confirm abnormal cell morphologies in peripheral blood (such as blasts, abnormal lymphocytes, immature granulocytes), thereby improving the accuracy in the auxiliary diagnosis of malignant hematological diseases [20, 21].

ML models have been used to assist in diagnosing different malignant hematological diseases or predicting their prognoses. Most of these models include clinical data from different sources, such as imaging, laboratory tests, or admission-related records [22–25]. In contrast, this study established ML models based on blood cell parameters only to screen for malignant hematological diseases because blood cell analysis is the most readily available, least expensive, and less impacted examination, with less biological variability than other tests [26]. This study selected the best ML model mainly by comparing the performance among the eight constructed ML models.

The eight ML models were compared in terms of discriminability (AUC, accuracy, precision, recall, specificity and F1-score), calibration (plots and Platt Scaling), and decision curve analysis. To evaluate the models from different perspectives, this study selected the best thresholds in these three aspects to observe the basic performance of the ML models. The results showed that all models had good AUCs at different thresholds (0.83–0.91), Among these models, the ANN, RF, and GBDT models had the highest AUC values and highest net clinical benefit according to decision curve analysis. The ANN model also had the highest accuracy and F1 score as well as the smallest range when selecting the optimal threshold; that is, when the ANN model threshold was 0.5, had the best accuracy, sensitivity, specificity and precision. In terms of calibration ability, the SVM and MLP models performed well, but their accuracy was slightly inferior to that of the ANN model. We found a risk of overconfidence in the calibration curve of the ANN model, so we calibrated it with Platt scaling [27]; the recalibrated ANN model had better performance. Among previous studies [13], Syed-Abdul also chose ANN as the model architecture for screening malignant hematological diseases, but they only focused on classical indicators (AUC, sensitivity, specificity, etc.) and did not provide calibration information. They also provided no further analyses of the black-box nature of the ANN model. Many ML models (e.g. SVM) produce raw scores that are not actual probabilities. Platt Scaling converts these scores to probabilities, ensuring that they fall within the range [0,1]. The probability improves the interpretability of the model, making it more intuitive and understandable, also providing more reliable confidence estimates. And it facilitates comparisons between multiple models.

We used the calibrated ANN model to identify malignant hematological diseases among the 528 samples in the validation set, which yielded 45 false-negative samples. On further analysis, we found that 22 patients in the validation set had lymphoma. Lymphoma is a group of heterogeneous solid tumors of the immune system; it has long been reported that the complete blood count in these patients, even those with aggressive lymphoma, is still normal, and conditions reflecting abnormal complete blood counts, such as anemia, thrombocytopenia, and leukopenia/lymphocytosis, are more suggestive of possible complications of lymphoma [28, 29]. Therefore, it is difficult to construct an ANN model based on peripheral blood cell parameters only for lymphoma patients without complications, limiting their utility to a certain extent. Indeed, lymphomas demonstrating insubstantial peripheral blood changes can be effectively identified with the model. Eight patients were in treatment for malignant hematological diseases, such as chemotherapy, and their peripheral blood cell characteristics were not obviously affected. Thirty false-positive samples were also identified, 8 of the 15 anemia patients had aplastic anemia (AA). The model constructed in this study also used features such as blasts, but the levels were still indistinguishable among individual samples, [30].

In addition, this study further explained the black-box mechanisms of the models for screening of malignant hematological diseases with SHAP analysis. We found that PLT, blasts and PCT were the most important features in the constructed ANN model. Hematologic neoplasms are malignant diseases originating from hematopoietic cells, mostly due to the proliferation of abnormal cells in the bone marrow that accumulate and inhibit normal hematopoiesis [3]. Doctors often suspect malignant hematologic diseases when blasts are present in the peripheral blood. In the WHO guidelines, blasts greater than 20% can be used as a diagnostic criterion for acute leukemia [3]. This is consistent with the results of SHAP analysis in this model, and the percentage of blasts has an important role in our model, when more blasts are present, the more it correlates with malignant hematologic diseases. Platelets are nucleated fragments derived from mature megakaryocytes in the bone marrow and are the main effector cells involved in the hemostatic nuclear thrombosis [31]. Detection of platelet parameters in patients with malignant hematologic diseases can indirectly reflect the changes in bone marrow function in patients with different stages of malignant hematologic diseases, which can help in the diagnosis of the disease and evaluation of the efficacy of the treatment [31]. Our model SHAP analysis showed that the most relevant blood cell parameters are PLT and PCT, where PCT is obtained by multiplying PLT and MPV, which is influenced by the number and size of platelets and usually coincides with changes in PLT [32]. Previous studies have concluded that this may be due to changes in blood rheological properties in patients with hematological diseases, thereby affecting platelet function and distribution, but the specific mechanism of action has not yet been elucidated [13, 33]. In addition to these three, other important features in the model, such as neutral lobulated granulocytes and RDW, were also considered significant in previous studies differentiating MDS patients from non-MDS patients. Studies have focused on neutrophil structural dispersion (Neu-WX), erythrocyte size, and hemoglobin-containing heterogeneity when investigating the blood cell characteristics of MDS patients [10, 34]. We found that the same blood cell parameters had different feature importance in different models in different studies, which may be mainly related to differences in the distributions of the collected samples and the ML models constructed. Therefore, external validation in larger datasets is an essential step to verifying the ML model under study for clinical applicability [15, 25, 35].

There are limitations to this study in that, apart from the initial patient population, it lacked an external validation phase and did not consider factors that could potentially influence the model, such as the effect of treatment regimens on the model. These should be carefully analyzed in further studies. In addition, hematology analyzers produced by different manufacturers are based on different principles and threshold output parameters, which severely limits the widespread use of ML models based on blood cell parameters in clinical practice. This problem must be addressed if these ML models are to be generalized [36]. Finally, we must recognize that ML models can only serve to assist physicians in the initial screening of malignant hematologic diseases. The strength of this model lies in the incorporation and processing of high-dimensional information on patients' blood cell parameters through ML models [37], but not every patient can be fully predicted by ML models, and risks such as data sparsity, multicollinearity, and overfitting can be expected [35, 38], and ultimately, it is still the physicians who will take the next steps in clinical diagnosis and treatment. Although the ML model constructed based on blood cell parameters in this study has some limitations in screening malignant hematological diseases, the complete diagnostic process of acute leukemia, which includes cytomorphology, immunophenotyping, cytogenetics, and molecular biology, takes at least 3 days to complete, and in the future, the model will be applied to hematology analyzers, the convenience of blood cell analysis as the first screening test for patients admitted to the hospital, and its. The convenience and time-saving nature of blood cell analysis as the first screening test for patients admitted to the hospital is beyond doubt, and it is of particular importance for rural and community-based hospitals that lack advanced diagnostic equipment.

## Conclusions

In this study, eight ML models were constructed by using blood cell parameters, and their performance in screening malignant hematological diseases was comprehensively evaluated. Compared with the other ML models, the ANN model achieved higher accuracy and better performance. This study shows that ML models based on blood cell analysis parameters can screen patients for malignant hematological diseases inexpensively and highly efficiently. Especially in the future, this ANN model combining with blood cell analyzers in the primary hospitals that lack comprehensive diagnosis and treatment measures will help the patients to receive the diagnosis and treatment of malignant hematological diseases as early as possible.

## Supplementary Information

Supplementary Material 1.

## Data Availability

Data cannot be shared openly but are available on request from authors.

## Abbreviations

ANN: Artificial Neural Network
AUC: Area under the Receiver Operating Characteristic Curve
WHO: World Health Organization
SF cube: Scatter fluorescence cube
NCCN: National Comprehensive Cancer Network
ML: Machine Learning
MDS: Myelodysplastic Syndromes
LR: Logistic Regression
NB: Naïve Bayes
KNN: K-Nearest Neighbor
SVM: Support Vector Machines
RF: Random Forest
MLP: Multi-Layer Perceptron
GBDT: Gradient Boosting Decision Tree
CPD: Cell Population Data
TRIPOD: Transparent Reporting of a Multivariable Prediction Model for Individual Prognosis or Diagnosis
ROC: Receiver Operating Characteristic Curve
ICD-10: International Classification of Diseases
DCA: Decision Curve Analysis
SHAP: Shapley Additive Explanation

## References

[1] H. Sung, J. Ferlay, R.L. Siegel, M. Laversanne, I. Soerjomataram, A. Jemal, F. Bray, Global Cancer Statistics 2020: GLOBOCAN Estimates of Incidence and Mortality Worldwide for 36 Cancers in 185 Countries, CA: a cancer journal for clinicians 71(3) (2021) 209–249. 10.3322/caac.21660 33538338

[2] H. Gelband, R. Sankaranarayanan, C.L. Gauvreau, S. Horton, B.O. Anderson, F. Bray, J. Cleary, A.J. Dare, L. Denny, M.K. Gospodarowicz, S. Gupta, S.C. Howard, D.A. Jaffray, F. Knaul, C. Levin, L. Rabeneck, P. Rajaraman, T. Sullivan, E.L. Trimble, P. Jha, Costs, affordability, and feasibility of an essential package of cancer control interventions in low-income and middle-income countries: key messages from Disease Control Priorities, 3rd edition, Lancet (London, England) 387(10033) (2016) 2133–2144. 10.1016/S0140-6736(15)00755-2 26578033

[3] Vardiman JW. The World Health Organization (WHO) classification of tumors of the hematopoietic and lymphoid tissues: an overview with emphasis on the myeloid neoplasms. Chem Biol Interact. 2010;184(1–2):16–20. 19857474 10.1016/j.cbi.2009.10.009

[4] Arber DA, Orazi A, Hasserjian R, Thiele J, Borowitz MJ, Le Beau MM, Bloomfield CD, Cazzola M, Vardiman JW. The 2016 revision to the World Health Organization classification of myeloid neoplasms and acute leukemia. Blood. 2016;127(20):2391–405. 27069254 10.1182/blood-2016-03-643544

[5] N.C.C. Network, NCCN Clinical Practice Guidelines in Oncology-Acute lymphoblastic leukemia (2020 Version II). (2020).

[6] Z. He, G. Shu, H. Lu, Application of SF-Cube 2.0 Technology in Platelet Count in Patients with EDTA-Dependent Pseudothrombocytopenia, Clinical laboratory 67(6) (2021). 10.7754/Clin.Lab.2020.201021 34107626

[7] N. Khongjaroensakun, N. Chaothai, L. Chamchomdao, K. Suriyachand, K. Paisooksantivatana, White blood cell differentials performance of a new automated digital cell morphology analyzer: Mindray MC-80, International journal of laboratory hematology (2023). 10.1111/ijlh.14119 37338111

[8] Aguirre U, Urrechaga E. Diagnostic performance of machine learning models using cell population data for the detection of sepsis: a comparative study. Clin Chem Lab Med. 2023;61(2):356–65. 36351434 10.1515/cclm-2022-0713

[9] R.Z. Haider, I.U. Ujjan, N.A. Khan, E. Urrechaga, T.S. Shamsi, Beyond the In-Practice CBC: The Research CBC Parameters-Driven Machine Learning Predictive Modeling for Early Differentiation among Leukemias, Diagnostics (Basel, Switzerland) 12(1) (2022). 10.3390/diagnostics12010138 PMC8774626 35054304

[10] Zhu J, Lemaire P, Mathis S, Ronez E, Clauser S, Jondeau K, Fenaux P, Adès L, Bardet V. Machine learning-based improvement of MDS-CBC score brings platelets into the limelight to optimize smear review in the hematology laboratory. BMC Cancer. 2022;22(1):972. 36088307 10.1186/s12885-022-10059-8 PMC9464379

[11] Hwang SM, Nam Y. Complete blood count and cell population data parameters from the Abbott Alinity hq analyzer are useful in differentiating myelodysplastic syndromes from other forms of cytopenia. Int J Lab Hematol. 2022;44(3):468–76. 34877795 10.1111/ijlh.13777

[12] Moor M, Banerjee O, Abad ZSH, Krumholz HM, Leskovec J, Topol EJ, Rajpurkar P. Foundation models for generalist medical artificial intelligence. Nature. 2023;616(7956):259–65. 37045921 10.1038/s41586-023-05881-4

[13] Syed-Abdul S, Firdani RP, Chung HJ, Uddin M, Hur M, Park JH, Kim HW, Gradišek A, Dovgan E. Artificial Intelligence based Models for Screening of Hematologic Malignancies using Cell Population Data. Sci Rep. 2020;10(1):4583. 32179774 10.1038/s41598-020-61247-0 PMC7075908

[14] Gould MK, Huang BZ, Tammemagi MC, Kinar Y, Shiff R. Machine Learning for Early Lung Cancer Identification Using Routine Clinical and Laboratory Data. Am J Respir Crit Care Med. 2021;204(4):445–53. 33823116 10.1164/rccm.202007-2791OC

[15] Moons KG, Altman DG, Reitsma JB, Ioannidis JP, Macaskill P, Steyerberg EW, Vickers AJ, Ransohoff DF, Collins GS. Transparent Reporting of a multivariable prediction model for Individual Prognosis or Diagnosis (TRIPOD): explanation and elaboration. Ann Intern Med. 2015;162(1):W1-73. 25560730 10.7326/M14-0698

[16] Huang Y, Li W, Macheret F, Gabriel RA, Ohno-Machado L. A tutorial on calibration measurements and calibration models for clinical prediction models. Journal of the American Medical Informatics Association : JAMIA. 2020;27(4):621–33. 32106284 10.1093/jamia/ocz228 PMC7075534

[17] Barnes PW, McFadden SL, Machin SJ, Simson E. The international consensus group for hematology review: suggested criteria for action following automated CBC and WBC differential analysis. Laboratory hematology : official publication of the International Society for Laboratory Hematology. 2005;11(2):83–90. 16024331

[18] Mezzatesta S, Torino C, Meo P, Fiumara G, Vilasi A. A machine learning-based approach for predicting the outbreak of cardiovascular diseases in patients on dialysis. Comput Methods Programs Biomed. 2019;177:9–15. 31319965 10.1016/j.cmpb.2019.05.005

[19] McCabe B, Liberante F, Mills KI. Repurposing medicinal compounds for blood cancer treatment. Ann Hematol. 2015;94(8):1267–76. 26048243 10.1007/s00277-015-2412-1 PMC4488459

[20] Zini G, Cantelli F, Scavone F, Barbagallo O, Ciminello A. Hematological performance of a last generation automated blood cell counter: The Mindray BC-6800 Plus. Int J Lab Hematol. 2020;42(4):439–49. 32343494 10.1111/ijlh.13218

[21] Xing Y, Liu X, Dai J, Ge X, Wang Q, Hu Z, Wu Z, Zeng X, Xu D, Qu C. Artificial intelligence of digital morphology analyzers improves the efficiency of manual leukocyte differentiation of peripheral blood. BMC Med Inform Decis Mak. 2023;23(1):50. 36991420 10.1186/s12911-023-02153-z PMC10061886

[22] Li H, Xu C, Xin B, Zheng C, Zhao Y, Hao K, Wang Q, Wahl RL, Wang X, Zhou Y. (18)F-FDG PET/CT Radiomic Analysis with Machine Learning for Identifying Bone Marrow Involvement in the Patients with Suspected Relapsed Acute Leukemia. Theranostics. 2019;9(16):4730–9. 31367253 10.7150/thno.33841 PMC6643435

[23] Moraes LO, Pedreira CE, Barrena S, Lopez A, Orfao A. A decision-tree approach for the differential diagnosis of chronic lymphoid leukemias and peripheral B-cell lymphomas. Comput Methods Programs Biomed. 2019;178:85–90. 31416565 10.1016/j.cmpb.2019.06.014

[24] A. Nazha, R. Komrokji, M. Meggendorfer, X. Jia, N. Radakovich, J. Shreve, C.B. Hilton, Y. Nagata, B.K. Hamilton, S. Mukherjee, N. Al Ali, W. Walter, S. Hutter, E. Padron, D. Sallman, T. Kuzmanovic, C. Kerr, V. Adema, D.P. Steensma, A. Dezern, G. Roboz, G. Garcia-Manero, H. Erba, C. Haferlach, J.P. Maciejewski, T. Haferlach, M.A. Sekeres, Personalized Prediction Model to Risk Stratify Patients With Myelodysplastic Syndromes, Journal of clinical oncology : official journal of the American Society of Clinical Oncology 39(33) (2021) 3737–3746. 10.1200/JCO.20.02810 PMC8601291 34406850

[25] Radakovich N, Nagy M, Nazha A. Machine learning in haematological malignancies. The Lancet Haematology. 2020;7(7):e541–50. 32589980 10.1016/S2352-3026(20)30121-6

[26] Cembrowski GS, Clarke G. Quality control of automated cell counters. Clin Lab Med. 2015;35(1):59–71. 25676372 10.1016/j.cll.2014.10.006

[27] Walsh CG, Sharman K, Hripcsak G. Beyond discrimination: A comparison of calibration methods and clinical usefulness of predictive models of readmission risk. J Biomed Inform. 2017;76:9–18. 29079501 10.1016/j.jbi.2017.10.008 PMC5716927

[28] Paquin AR, Oyogoa E, McMurry HS, Kartika T, West M, Shatzel JJ. The diagnosis and management of suspected lymphoma in general practice. Eur J Haematol. 2023;110(1):3–13. 36093749 10.1111/ejh.13863 PMC10042228

[29] Storck K, Brandstetter M, Keller U, Knopf A. Clinical presentation and characteristics of lymphoma in the head and neck region. Head Face Med. 2019;15(1):1. 30606206 10.1186/s13005-018-0186-0 PMC6317257

[30] Kimura K, Tabe Y, Ai T, Takehara I, Fukuda H, Takahashi H, Naito T, Komatsu N, Uchihashi K, Ohsaka A. A novel automated image analysis system using deep convolutional neural networks can assist to differentiate MDS and AA. Sci Rep. 2019;9(1):13385. 31527646 10.1038/s41598-019-49942-z PMC6746738

[31] L. Zhang, J. Liu, X. Qin, W. Liu, Platelet-Acute Leukemia Interactions, Clinica chimica acta; international journal of clinical chemistry 536 (2022) 29–38. 10.1016/j.cca.2022.09.015 36122665

[32] Asare R, Opoku-Okrah C, Danquah KO, Opare-Sem O, Addai-Mensah O, Gyamfi D, Amponsah FA, Afriyie EY, Duneeh RV, Ofosu DN, Frimpong M. Expression of platelet parameters and platelet membrane glycoproteins in childhood Burkitt lymphoma. Leuk Res. 2019;84: 106189. 31326577 10.1016/j.leukres.2019.106189

[33] Vinholt PJ. The role of platelets in bleeding in patients with thrombocytopenia and hematological disease. Clin Chem Lab Med. 2019;57(12):1808–17. 31465290 10.1515/cclm-2019-0380

[34] Raess PW, van de Geijn GJ, Njo TL, Klop B, Sukhachev D, Wertheim G, McAleer T, Master SR, Bagg A. Automated screening for myelodysplastic syndromes through analysis of complete blood count and cell population data parameters. Am J Hematol. 2014;89(4):369–74. 24276948 10.1002/ajh.23643

[35] Shouval R, Fein JA, Savani B, Mohty M, Nagler A. Machine learning and artificial intelligence in haematology. Br J Haematol. 2021;192(2):239–50. 32602593 10.1111/bjh.16915

[36] Seghezzi M, Buoro S, Previtali G, Moioli V, Manenti B, Simon-Lopez R, Ottomano C, Lippi G. A Preliminary Proposal for Quality Control Assessment and Harmonization of Leukocytes Morphology-structural Parameters (cell Population Data Parameters). Journal of medical biochemistry. 2018;37(4):486–98. 30584409 10.2478/jomb-2018-0005 PMC6298477

[37] Kurtz DM, Esfahani MS, Scherer F, Soo J, Jin MC, Liu CL, Newman AM, Dührsen U, Hüttmann A, Casasnovas O, Westin JR, Ritgen M, Böttcher S, Langerak AW, Roschewski M, Wilson WH, Gaidano G, Rossi D, Bahlo J, Hallek M, Tibshirani R, Diehn M, Alizadeh AA. Dynamic Risk Profiling Using Serial Tumor Biomarkers for Personalized Outcome Prediction. Cell. 2019;178(3):699-713.e19. 31280963 10.1016/j.cell.2019.06.011 PMC7380118

[38] Altman N, Krzywinski M. The curse(s) of dimensionality. Nat Methods. 2018;15(6):399–400. 29855577 10.1038/s41592-018-0019-x

