# Supplementary material for: A novel method for screening malignant hematological diseases by constructing an optimal machine learning model based on blood cell parameters
Source: BMC Med Inform Decis Mak. 2025 Feb 11;25:72. doi: 10.1186/s12911-025-02892-1 (PMC11816569; doi:10.1186/s12911-025-02892-1)
Supplement: Supplementary file 1 — Supplementary Material 1. [file 12911_2025_2892_MOESM1_ESM.docx]

Supplementary Table 1. 26 Blood Cell Parameters in Machine Learning Models

| NO | Parameters | Full Name | Instrument |
| --- | --- | --- | --- |
| 1 | PLT_I | Platelet count-Impedance channel | BC-7500CRP |
| 2 | PCT | Plateletcrit | BC-7500CRP |
| 3 | blast | Blast Cells percentage | MC-80 |
| 4 | RDW-SD | Red Blood Cell Volume Distribution Width–Standard Deviation | BC-7500CRP |
| 5 | RBC | Red Blood Cell count | BC-7500CRP |
| 6 | HGB | Hemoglobin Concentration | BC-7500CRP |
| 7 | MCHC | Mean Corpuscular Hemoglobin Concentration | BC-7500CRP |
| 8 | WBC | White Blood Cell count | BC-7500CRP |
| 9 | segmented neutrophils | Segmented Neutrophils percentage | MC-80 |
| 10 | TNC-N | Total nucleated cell count - WNB | BC-7500CRP |
| 11 | primative cells | Primative Cells | MC-80 |
| 12 | neutrophils | Neutrophils percentage | MC-80 |
| 13 | HCT | Hematocrit | BC-7500CRP |
| 14 | Eos% | Eosinophisl percentage | BC-7500CRP |
| 15 | RDW-CV | Red Blood Cell Volume Distribution Width–Coefficient of Variation | BC-7500CRP |
| 16 | IMG% | Immature Granulocyte percentage | BC-7500CRP |
| 17 | Mon% | Monocytes percentage | BC-7500CRP |
| 18 | basophils | Basophils percentage | MC-80 |
| 19 | MCV | Mean Corpuscular Volume | BC-7500CRP |
| 20 | Neu% | Neutrophils percentage | BC-7500CRP |
| 21 | MPV | Mean Platelet Volume | BC-7500CRP |
| 22 | Lym% | Lymphocytes percentage | BC-7500CRP |
| 23 | immature granulocytes | Immature Granulocytes percentage | MC-80 |
| 24 | Baso% | Basophils percentage | BC-7500CRP |
| 25 | monocytes | Monocytes percentage | MC-80 |
| 26 | abnormal lymphocytes | Abnormal Lymphocytes percentage | MC-80 |
